# Kahook Dual Blade Goniotomy Outcomes in the Underserved Dominican Republic Black and Afro-Latinx Population

**DOI:** 10.3390/jcm14072201

**Published:** 2025-03-24

**Authors:** Cristos Ifantides, Hernan Bejar, Jennifer Patnaik, Erin Sieck, Mina Pantcheva, Cara Capitena Young, Margarita Arbaje, William McCollum

**Affiliations:** 1 Department of Ophthalmology, University of Colorado Anschutz Medical Campus, Aurora, CO 80045, USA; cristos.ifantides@cuanschutz.edu (C.I.); jennifer.patnaik@cuanschutz.edu (J.P.); mina.pantcheva@cuanschutz.edu (M.P.);; 2 Tyson Eye, Cape Coral, FL 33904, USA; 3 Department of Ophthalmology, Hospital Dr. Elias Santana, Santo Domingo 10802, Dominican Republic; hernan_vbc@hotmail.com (H.B.); marbaje@hotmail.com (M.A.); wmhtrabajo@gmail.com (W.M.); 4 Instituto Tecnológico de Santo Domingo—INTEC, Santo Domingo 10602, Dominican Republic

**Keywords:** MIGS, glaucoma, black population, Caribbean, goniotomy

## Abstract

**Background/Objectives:** Black and Afro-Latinx communities have a higher propensity for more-severe glaucoma at a younger age. This study aimed to use the Kahook Dual Blade (KDB) to treat all glaucoma subtypes and severity levels in this historically underserved community. **Materials and Methods:** This study involved a retrospective analysis of surgical case records, with follow-up through 20 months. The subjects were glaucoma patients at Hospital Elias Santana in Santo Domingo, Dominican Republic. Inclusion criteria were age >18 years and a minimum follow-up of 1 year after surgery. All glaucoma subtypes and disease severities were included, including previous glaucoma procedures. Sequential patients undergoing KDB goniotomy alone or in combination with phacoemulsification cataract surgery were assessed. IOP data and number of medications were collected at specific time points: baseline, postoperative day 1, postoperative week 1, and postoperative months 1, 3, 6, 12, 16, and 20. Surgical outcome was determined using IOP and number of medications. Success was defined as either a 20% or more reduction in IOP or a decrease in at least one topical therapy. Recorded postoperative complications were hyphema, ocular hypertension, and need for additional glaucoma surgery. **Results:** A total of 90 eyes from 90 patients were included. A total of 100% of the patient population was Black or Afro-Latinx. The most common glaucoma subtype was primary open-angle (76.7%). Most of the eyes had severe glaucoma (53.3%). The mean preoperative baseline IOP was 20.5 mmHg. The mean postoperative IOP from all time points ranged from 12.9 to 13.5 mmHg (all time points were significantly lower than baseline IOP, *p* < 0.0001). A mean reduction in IOP percent of at least 31.5% was seen at every time point. There was a mean reduction of two medications by postoperative month 20. Surgical success was achieved in 95.6% of patients at postoperative month 1 and remained high throughout the study period (95.4% at month 20). **Conclusions:** KDB goniotomy achieved successful IOP and medication reduction across all levels of glaucoma severity. Surgical success rates were maintained to 20 months. While MIGS has historically been used as an intervention in mild-to-moderate glaucoma, our study results show that the KDB can play a significant role in all stages of glaucoma, including severe. MIGS should be considered as a favorable intervention in all disease severities in Black and Afro-Latinx communities around the world.

## 1. Introduction

Glaucoma and cataracts are the leading causes of blindness globally [1]. There is a high prevalence of glaucoma in Black and Afro-Latinx communities with a higher propensity for more-severe disease at a younger age compared with Caucasian [2]. Reducing intraocular pressure (IOP) remains the only modifiable risk factor that has been proven to slow disease progression for glaucoma [3].

Treatment options include topical eye drops to reduce IOP and various surgical procedures. Advanced surgical treatments for severe and refractory glaucoma include filtering surgery, glaucoma drainage devices (GDDs), and cyclodestructive procedures. These surgeries have an unfavorable safety profile but may provide a more effective IOP reduction than less invasive alternatives, such as drops or selective laser trabeculoplasty [4]. In addition, these more invasive procedures may limit treatment options later in the disease process.

Over the last decade, there has been an increasing interest in micro-invasive glaucoma surgery (MIGS), broadening the options for surgical interventions for glaucoma. MIGSs are a group of surgical techniques that share the following properties: ab interno approach, minimal trauma to the target tissue, modest efficacy, improved safety profile, and faster recovery times compared with the more invasive glaucoma procedures [5].

A popular target of several MIGS devices is the trabecular meshwork, as it is the primary site of resistance of aqueous outflow [6]. The Kahook Dual Blade (KDB, New World Medical Inc., Rancho Cucamonga, CA, USA) is a device which aims to perform a safe and efficacious goniotomy with trabecular-meshwork (TM) excision [7]. The device is designed with a taper at the tip to allow for smooth entry of the blade into Schlemm’s canal and parallel blades that cut the trabecular meshwork with minimal damage to surrounding tissues [8]. This is in contrast to other goniotomy devices that are incisional and do not completely remove trabecular meshwork, which may lead to scarring and decreased long-term efficacy. The KDB can be used in combination with cataract extraction or used as a standalone procedure. There are several published studies demonstrating the safety and efficacy of the KDB [9,10,11]. It remains a popular MIGS for glaucoma specialists given the efficacy and absence of an implant left within the eye.

Given moderate efficacy, unknown length of effective IOP reduction, and lack of comparative MIGS studies, newer glaucoma procedures are most often utilized in mild-to-moderate glaucoma [12]. There is published data from several retrospective reviews showing the KDB can be efficacious at lowering IOP in later-stage glaucoma [13]. Hirabayashi et al. demonstrated an IOP less than 15 mmHg in 64.3% of their severe-glaucomatous eyes at 6 months following phacoemulsification combined with goniotomy [14]. Similarly, Bravetti et al. showed mean IOP decreased from 18.1 ± 5.0 mmHg at baseline to 14.8 ± 3.7 mmHg at 12 months in a small study of severe-glaucoma eyes [15]. One of the limitations to both of these studies is that the majority of the patients identified their race as Caucasian [11]. Therefore, the generalizability to other racial/ethnic groups is not well established.

“Afro-Latinx” people are persons who identify their ethnicity as Latino and their race as Black. Black and Afro-Latinx patients are often underrepresented in glaucoma studies, despite having more-severe disease. There is a suspected greater rate of scarring in response to filtration surgeries among Afro-Latinx patients, hence the added interest in evaluating their response to MIGS and avoidance of a bleb-forming procedure [13]. Laroche et al. looked at KDB goniotomy with and without phacoemulsification in the Black and Afro-Latinx population in the United States. Their study demonstrated a successful reduction in IOP at 6 months in both groups. While the severity of glaucoma was not reported, the baseline mean MD was −8.63 dB in the combined phacoemulsification and goniotomy group and was −15.27 dB in the standalone KDB group. These reported baseline MDs on Humphrey visual fields suggest the patients had moderate-to-severe glaucoma. Their study ended at 6 months, so the long-term IOP reduction is unknown.

To our knowledge, no study has specifically examined the long-term (greater than 1 year) efficacy of KDB goniotomy combined with cataract surgery in the Black and Afro-Latinx patient population in the Dominican Republic. In addition, the majority of our patients were classified as having severe-stage glaucoma, which is often excluded in other MIGS studies.

## 2. Methods

Written institutional review board (IRB)/ethics committee approval was obtained through the Elias Santana Hospital Bioethics Committee in Santo Domingo, Dominican Republic. The described research herein adhered to the tenets of the Declaration of Helsinki. A retrospective chart review was completed on sequential patients undergoing KDB goniotomy alone or in combination with phacoemulsification cataract surgery at Hospital Elias Santana in Santo Domingo, Dominican Republic, between February and June 2019. Inclusion criteria were age >18 years old and a minimum follow-up of at least 1 month after surgery. All glaucoma subtypes and disease severities (mild, moderate, and severe) were included. Patients were included regardless of previous laser treatment but were excluded if they had previous glaucoma surgeries.

Demographics and ophthalmic conditions for each patient were recorded. IOP data and number of medications were collected at the following time points: baseline, postoperative day 1, postoperative week 1, and postoperative months 1, 3, 6, 12, 16, and 20. The following postoperative complications were recorded for analysis: hyphema, IOP spike, and need for additional glaucoma surgery. Hyphema was defined as visible layered blood in the anterior chamber. IOP spike was defined as an increase in IOP > 10 mmHg from baseline or any IOP over 30 mmHg. Time points after another glaucoma surgery were excluded. The definition of success was defined as an IOP reduction of at least 20% or reduction in at least one glaucoma medication from baseline level compared with each follow-up time period as previously outlined in other MIGS studies.

### 2.1. Kahook Dual Blade Surgical Technique

Kahook Dual Blade was performed after phacoemulsification cataract surgery with intraocular lens implantation in the bag. The microscope was tilted 30 degrees toward the surgeon while the patient’s head was rotated 45 degrees away from the surgeon. The primary temporal 2.2 mm clear-corneal wound from the cataract surgery was used for the Kahook Dual Blade procedure. Cohesive ProVisc viscoelastic (Alcon, Fort Worth, TX, USA) was injected into the anterior chamber. A Swan Jacob gonioprism (Ocular instruments) was placed on the corneal surface with dispersive Viscoat viscoelastic (Alcon, Fort Worth, TX, USA) with the left hand to visualize the angle structures. The right hand was used to hold the KDB and introduce it through the main wound until it reached the trabecular meshwork. Once located on the trabecular meshwork, it was placed parallel to the plane of the iris, resting on the trabecular meshwork. The leading edge was pushed forward, penetrating the trabecular meshwork. Once in Schlemm’s canal, constant pressure was applied, moving forward from right to left in the direction of the tip of the blade, advancing 3 clock hours from the most distal side towards the center. The KDB was then rotated 180 degrees inside the anterior chamber, and the procedure was repeated from left to right, advancing 1 clock hour from the distal side to the center so that the two cuts were joined for a total of approximately 4 clock hours. This is known as the “outside-in” KDB technique. The KDB was then retired from the anterior chamber, and the patient’s head and the microscope were brought to the original position. Viscoelastic was removed along with the trabecular-meshwork strip. Carbachol was instilled into the anterior chamber to constrict the pupil. Moxifloxacin (Vigamox, Alcon, Fort Worth, TX, USA) was instilled into the anterior chamber. All wounds were hydrated and checked to ensure watertight closure. And, an air bubble was inserted in the anterior chamber to finish each case. Postoperatively, patients were started on a standard regimen of 1% prednisolone acetate suspension and 0.5% moxifloxacin solution dosed six times a day for one week. Nepafenac 0.1% was dosed two times a day. After one week, moxifloxacin was discontinued, and the remaining two medications were tapered off over the following 4 weeks.

### 2.2. Statistical Analysis

IOP changes (%) were calculated at each time point using the difference between preoperative baseline IOP measurement minus postoperative time-point IOP, divided by preoperative baseline IOP. A linear model with generalized estimating equations using an exchangeable correlation structure was used to compare the mean IOP and number of medications pre- and postoperatively. IOP and medication burden were compared between preoperative baseline and postoperative time points. Sub-analyses were performed on patients with severe glaucoma for the primary outcomes of IOP and number of medications. Means, medians, ranges, and standard deviations are reported.

Surgical success was defined at each time point as IOP reduction and/or reduction in at least one glaucoma medication. Success rates and rates of complications are presented as basic frequencies and percentages. Best-corrected visual acuity (BCVA) was collected at baseline and the 12-month follow-up as Snellen and transformed to LogMAR values for statistical analyses.

## 3. Results

In total, 90 eyes from 90 patients were included in the analysis. A total of 87 eyes underwent KDB goniotomy combined with cataract surgery (phaco-KDB), and 3 eyes underwent KDB goniotomy only. No patients had prior glaucoma procedures, and the three undergoing standalone goniotomy had undergone prior phacoemulsification. Patient demographics for the entire study population are presented in Table 1. All the patient population were Black or Afro-Latinx, and 50% of the study population was female. The mean patient age was 65.3 years (SD 13.5; range 24–89). Multiple types of glaucoma were represented, with the most common subtypes being primary open-angle (76.7%) and pigmentary (15.6%). The majority of the eyes had severe glaucoma (53.3%). Two patients were excluded due to the need for further surgical intervention to manage ocular hypertension. Baseline mean BCVA was 0.806 (SD: 0.51), which improved to a mean of 0.490 (SD: 0.398) at the one-year follow-up.

### 3.1. Intraocular-Pressure Reduction

**Figure 1:**
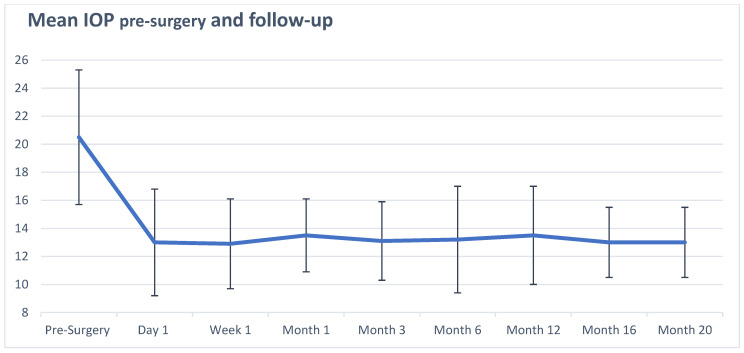
Mean IOP preoperative and at postoperative time points. This figure shows the mean IOP before and after surgical intervention. Mean values are indicated by the blue line. Bar lines represent standard deviations at each time point. *p*-value < 0.0001 at all-time points.

In Figure 1 and Table 2, we include the mean IOP for all patients during the 20-month follow-up. At each postoperative time point, there was a statistically significant reduction in mean IOP, with all *p*-values of <0.0001. The median preoperative baseline IOP was 20 mmHg, and the median postoperative IOP was 13 mmHg at all time points. The mean preoperative baseline IOP was 20.5 mmHg, and the mean postoperative IOP ranged from 12.9 to 13.5 mmHg. IOP reduction below 21 mmHg was achieved in 87 of 90 patients (96.7%) by postoperative day 1 and remained stable throughout the study period, with a final IOP < 21 mmHg seen in 88 of 88 included patients (100%) at the 20-month follow-up. Two patients required additional surgery during the study period for uncontrolled IOP. There was a mean reduction in IOP percent change of at least 31.5% at every time point. In the analysis of the sub-cohort of patients diagnosed with severe glaucoma, findings were virtually the same in regard to mean IOPs and significance (all *p* < 0.0001).

### 3.2. Medication Burden

**Figure 2:**
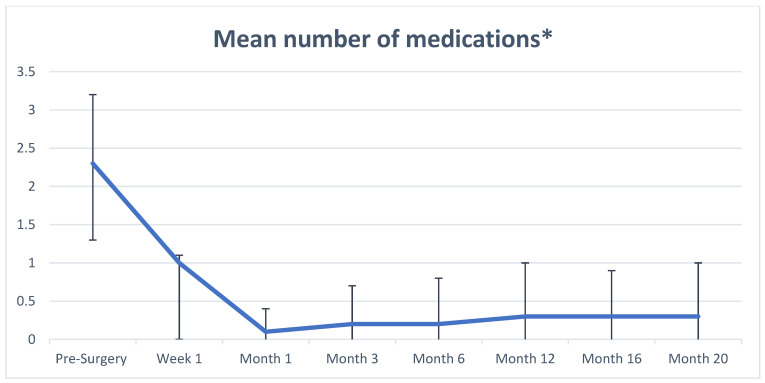
Mean number of medications preoperative and at postoperative time points. This figure shows the mean number of medications before and after surgical intervention. Mean values are indicated by the blue line. Bar lines represent standard deviations at each time point. * *p*-value < 0.0001 at all time points.

The mean numbers of medications at baseline and follow-up time points are shown in Figure 2 and Table 3. The preoperative baseline mean number of medications was 2.3. By postoperative month 1, the mean decreased to 0.1 and remained stable at 0.2 for month 6, then at 0.3 through month 20, representing an average reduction of two medications compared with the baseline. In the analysis of the sub-cohort of patients diagnosed with severe glaucoma, the findings were very similar, with a mean number of medications at baseline that was slightly higher at 2.8 and similar mean at 20 months postoperative of 0.3 (all *p* < 0.0001).

### 3.3. Surgical Success

Surgical success was defined as an IOP reduction of at least 20% or reduction in at least one glaucoma medication. Success rates were similar at all time points by gender and age. Success was 98.9% at postoperative month 1 and remained high throughout the study period (95.4% at month 20), as demonstrated in Table 4.

### 3.4. Complications

Table 5 displays complications by type of surgery. Complications included postoperative IOP spike and hyphema. Hyphema was seen in 4 of 90 patients (4.4%) and an IOP spike was seen in 3 of 90 patients. No patient had both hyphema and IOP spike. Two patients (2.2%) who had an IOP spike required additional glaucoma surgery and were eventually excluded from future analysis. All complications were encountered in the KDB/phaco group. Other complications found in the MIGS literature, such as corneal edema, glare, prolonged decreased vision, and endophthalmitis, were not found in this review.

## 4. Discussion

In this study, we demonstrate the safety and efficacy of KDB goniotomy in the Black and Afro-Latinx population in the Dominican Republic. KDB goniotomy achieved successful lowering of IOP and reduction in medication burden across all levels of glaucoma severity, with just over half of our patients being severe-stage. Surgical success rates were maintained out to the 20-month follow-up.

Comparing our data to prior KDB goniotomy studies, we see a similar percent reduction in IOP with prolonged follow-up. Sieck et al. in 2018 presented the first clinical data on KDB outcomes with and without phacoemulsification [13]. At 12 months, they demonstrated a mean 20.4% reduction in IOP. At 12 months, we demonstrated a mean 32% reduction in mean IOP. Comparing our data sets, we had a higher pre-surgical mean IOP, more severe-glaucoma patients, and a population of entirely Black and Afro-Latinx patients. Our improved success seen might reflect good response to trabecular-meshwork-based glaucoma surgery in Black and Afro-Latinx populations. Unfortunately, this patient population is underrepresented in other glaucoma surgical studies, so this correlation cannot be confirmed but should be an area of future studies.

While MIGS has historically been used as an intervention in mild-to-moderate glaucoma, our study results show that MIGS can play a significant role at all stages of glaucoma. This knowledge gives the ability for the glaucoma surgeon and patient to decide which approach is best for the patient. With improved safety profiles, MIGS can help significantly reduce the IOP in a broader range of patients than previously described. Of note, two patients did require additional glaucoma surgery for IOP control. Despite MIGS controlling IOP for the majority of patients, its limitations for all patients must be recognized.

Our data set also represented several subtypes of glaucoma, including pigmentary glaucoma, juvenile glaucoma, primary open-angle glaucoma, and chronic angle-closure glaucoma. We found similar success across our glaucoma subtypes, which agrees with prior published data showing success with trabecular-meshwork unroofing procedures and variable subtypes. We did not have a large enough data set to see if one glaucoma subtype responded superiorly; this could be an area of future studies.

Interestingly, our average age at time of surgery (65.3 years) is less than other published data [13]. This is likely due to the earlier onset of glaucoma in the Black and Latinx community, which made up our entire study population. With advancing age, trabecular meshwork and uveoscleral-outflow facility gradually decline [6]. Some of our success may be due to operating on younger eyes with better distal outflow pathways, but this supports the ideology to intervene on glaucoma patients at younger ages and still have surgical success. The benefit of less invasive interventions at younger ages is still yet to be determined.

The Baltimore Eye Study, published in 1991, showed a glaucoma prevalence rate of 4 percent among African Americans aged 50–59, with an increase to approximately 13 percent in African Americans aged 80–89. After correcting for age, African Americans had a three-to-four times higher chance of having glaucoma compared with their Caucasian counterparts [16]. Similarly, the Los Angeles Latino Eye Study published in 2004 found the prevalence of primary open-angle glaucoma to be similar to African Americans at around 5 percent [17]. There is no identified reason why Black and Latino populations are disproportionately impacted with glaucoma. This higher prevalence and fact that Black and Latino populations are routinely underrepresented in pivotal glaucoma studies are issues that need addressing. The Advanced Glaucoma Intervention Study (AGIS) was one of the few landmark trials studying more self-identified Black patients than White patients and investigated two randomly assigned surgical intervention sequences: argon-laser-trabeculoplasty (ALT)–trabeculectomy–trabeculectomy (ATT) or trabeculectomy–ALT–trabeculectomy (TAT). In this study, they found superior IOP reduction in the TAT protocols but worsening visual field loss compared with Black patients undergoing the ATT protocol. Based on the visual field loss, it was suggested to follow ALT as first-line treatment for Black patients with severe glaucoma [18]. Based on our data set, we see a favorable safety profile with no loss of BCVA and good IOP reduction with surgery, demonstrating a safe first-line intervention for severe-glaucoma patients of Black and Afro-Latinx races. Our results have implications in underserved communities of Black populations in North America, South America, Europe, and especially sub-Saharan Africa, where glaucoma has historically been underdiagnosed and undertreated due to access of care and the resource-intensive nature of glaucoma care [19].

In addition to favorable IOP reduction, our patients had few postoperative complications, with transient hyphema (4.4%) and ocular hypertension (3.3%) being the most common. There was no serious loss of vision or endophthalmitis cases. Two patients who also had IOP spikes required additional glaucoma surgical intervention during the study period and were eventually removed from statistical analysis. These rates seem comparable with that of other MIGS studies and show the continued safety of less invasive procedures.

The limitations of this study include the retrospective nature, small sample size, and limited follow-up to 20 months. This was a retrospective review, and patients were chosen by the surgeon to undergo goniotomy with or without phacoemulsification. This does present a bias for better angle anatomy to be selected for an angle-based procedure. The impact of preoperative gonioscopy grading and postoperative outcomes is not known. There is also bias in patients with higher IOP being chosen for this study, which may overinflate the precent reduction in IOP. This study was limited to 90 patients. To corroborate the success of goniotomy in Black and Afro-Latinx communities, we need to continue to expand our study size and have prolonged follow-up to strengthen these current findings. Lastly, we did not follow visual field data. This would be ideal to show that a lower IOP in severe-glaucoma patients may be associated with prevention of visual field progression.

This study demonstrates one of several MIGSs available on the market. We show that targeting the trabecular meshwork is an effective option for Black and Afro-Latinx patients, but this idea can be expanded when we are able to compare several types of trabecular-meshwork-based MIGSs. A prospective comparative trial with over 2 years of data would be ideal to adequately show the efficacy of goniotomy in this patient population.

In conclusion, the KDB goniotomy is a well-established procedure for glaucoma. We present success of this procedure in a study group of all Black and Afro-Latinx patients in the Dominican Republic with the majority with severe glaucoma. MIGS will continue to play an integral part of the surgical plan for patients, even in advancing disease, and should be considered a favorable intervention in Black and Afro-Latinx communities around the world.

## Figures and Tables

**Table 1:** Characteristics of Kahook Dual Blade study population, February 2019 to June 2019. This table outlines the demographics of the study population.

	N	%
Patients	90	
Eyes	90	
Gender		
Female	45	50.0%
Male	45	50.0%
Age, years		
Mean (SD)	65.3 (13.5)	
Median	66	
Range	24–89	
Eyes		
Left	44	48.9%
Right	46	51.1%
Glaucoma Severity		
Mild	8	8.9%
Moderate	34	37.8%
Severe	48	53.3%
Glaucoma Type		
CACG	4	4.4%
Juvenile	3	3.3%
POAG	69	76.7%
Pigmentary	14	15.6%
Type of Surgery		
KDB and Phaco	87	96.7%
KDB only	3	3.3%
Preop BCVA		
Mean (SD)	0.806 (0.51)	
Median	0.699	
Range	0–2.5	
Postop BCVA		
Mean (SD)	0.490 (0.48)	
Median	0.398	
Range	0–2.5	

**Table 2:** IOP before the surgery and at follow-up visits. This table shows the intraocular pressure at baseline and each follow-up. It also demonstrates the percent reduction in IOP after surgery.

	Preoperative	Day 1	Week 1	Month 1	Month 3	Month 6	Month 12	Month 16	Month 20
n	90	90	90	89	89	89	89	88	88
IOP									
Mean (SD)	20.5 (4.8)	13.0 (3.9)	12.9 (3.8)	13.5 (2.9)	13.1 (2.8)	13.2 (3.7)	13.5 (3.5)	13.0 (2.5)	13.0 (2.5)
Median	20	13	13	13	13	13	13	13	13
Range	11–36	5–29	5–34	7–28	7–22	8–39	6–29	7–21	8–19
*p*-value *	-	<0.0001	<0.0001	<0.0001	<0.0001	<0.0001	<0.0001	<0.0001	<0.0001
IOP									
<21	47 (52.2%)	87 (96.7%)	88 (97.8%)	88 (98.9%)	87 (97.8%)	87 (97.8%)	87 (97.8%)	87 (98.9%)	88 (100%)
21+	43 (47.8%)	3 (3.3%)	2 (2.2%)	1 (1.1%)	2 (2.2%)	2 (2.2%)	2 (2.2%)	1 (1.1%)	0 (0%)
Percent IOP Reduction									
Mean (SD)	-	−34.1 (22.6)	−34.5 (23.4)	−31.5 (18.8)	−33.9 (17.3)	−32.1 (26.6)	−32.0 (21.2)	−33.8 (18.5)	−34.4 (17.4)
Median		−36.2	−38.5	−34.6	−36.8	−35.7	−38.1	−39.1	−39.2
Range		−74, 46	−72, 79	−69, 47	−67, 27	−69, 144	−68, 54	−72, 23	−67, 12

* *p*-value compared with baseline.

**Table 3:** Medication burden before the surgery and at follow-up visits. This table outlines the number of medications before and after surgery at each follow-up.

	Preoperative	Day 1	Week 1	Month 1	Month 3	Month 6	Month 12	Month 16	Month 20
n	90	90	90	89	89	89	89	88	88
Number medications	2.3 (0.9)		1.0 (0.1)	0.1 (0.4)	0.2 (0.6)	0.2 (0.7)	0.3 (0.7)	0.3 (0.7)	0.3 (0.7)
Mean (SD)	3		1	0	0	0	0	0	0
Medianrange	0–4		1–2	0–3	0–3	0–3	0–3	0–3	0–3
*p*-value *		-	<0.0001	<0.0001	<0.0001	<0.0001	<0.0001	<0.0001	<0.0001
Decreased medications by at least one	-	-	74 (82.2%)	86 (96.6%)	84 (94.4%)	83 (94.3%)	81 (93.1%)	82 (93.2%)	82 (93.2%)
Not on any medications	2 (2.2%)	-	0 (0%)	83 (93.3%)	77 (86.5%)	74 (84.1%)	71 (81.6%)	72 (81.8%)	72 (81.8%)

* *p*-value compared with baseline.

**Table 4:** Success rate at follow-up for all patients. This table shows the percentage of patients with surgical success, defined as IOP reduction of at least 20% or reduction in at least one glaucoma medication at each follow-up visit.

	Pre-Surgery	Day 1	Week 1	Month 1	Month 3	Month 6	Month 12	Month 16	Month 20
Surgery success	-	-	86 (95.6%)	88 (98.9%)	87 (97.8%)	86 (97.7%)	86 (97.7%)	86 (97.7%)	84 (95.4%)

**Table 5:** Complications by type of surgery. This table compares complications seen with KDB goniotomy combined with phacoemulsification and KDB standalone.

	Total	KDB/Phaco	KDB Only
Total eyes	90	87	3
Complications			
Transient hyphema	4 (4.4%)	4 (4.6%)	0
Hypertension	3 (3.3%)	3 (3.4%)	0
Additional surgery	2 (2.2%)	2 (2.3%)	0

## Data Availability

Data are contained within the article.

## Abbreviations

KDB	Kahook Dual Blade
IOP	intraocular pressure
MIGS	minimally invasive glaucoma surgery
